# Advanced oxidation protein products induce pre‐osteoblast apoptosis through a nicotinamide adenine dinucleotide phosphate oxidase‐dependent, mitogen‐activated protein kinases‐mediated intrinsic apoptosis pathway

**DOI:** 10.1111/acel.12764

**Published:** 2018-04-16

**Authors:** Si‐Yuan Zhu, Jing‐Shen Zhuang, Qian Wu, Zhong‐Yuan Liu, Cong‐Rui Liao, Shi‐Gan Luo, Jian‐Ting Chen, Zhao‐Ming Zhong

**Affiliations:** ^1^ Department of Spinal Surgery Nanfang Hospital Southern Medical University Guangzhou China

**Keywords:** advanced oxidation protein products, apoptosis, osteoblast, osteoporosis, oxidative stress, reactive oxygen species

## Abstract

Osteoblast apoptosis contributes to age‐related bone loss. Advanced oxidation protein products (AOPPs) are recognized as the markers of oxidative stress and potent inducers of apoptosis. We have demonstrated that AOPP accumulation was correlated with age‐related bone loss. However, the effect of AOPPs on the osteoblast apoptosis still remains unknown. Exposure of osteoblastic MC3T3‐E1 cells to AOPPs caused the excessive generation of reactive oxygen species (ROS) by activating nicotinamide adenine dinucleotide phosphate (NADPH) oxidases. Increased ROS induced phosphorylation of mitogen‐activated protein kinases (MAPKs), which subsequently triggered intrinsic apoptosis pathway by inducing mitochondrial dysfunction, endoplasmic reticulum stress, and Ca^2+^ overload and eventually leads to apoptosis. Chronic AOPP loading in aged Sprague‐Dawley rats induced osteoblast apoptosis and activated NADPH oxidase signaling cascade, in combination with accelerated bone loss and deteriorated bone microstructure. Our study suggests that AOPPs induce osteoblast apoptosis by the NADPH oxidase‐dependent, MAPK‐mediated intrinsic apoptosis pathway.

## 1 INTRODUCTION

Osteoporosis is a chronic, progressive disease characterized by low bone mass, bone deterioration, and decreased bone strength, with a consequent increase in bone fragility and susceptibility to fracture (Reginster & Burlet, 2006). The prevalence of osteoporosis increases markedly with age. An epidemiological survey in America targeted aged 50 years or older showed that 13%–18% had osteoporosis and 37%–50% had osteopenia (Looker et al., 1997). Nowadays, one in three women over the age of 50 years and one in five men at the same age will experience osteoporotic fractures in their lifetime (Brown, 2017). Osteoporosis is increasingly being recognized as an important cause of morbidity and mortality in the elderly population (Cummings & Melton, 2002; Jilka & O'Brien, 2016).

Bone remodeling depends on the spatial and temporal coupling between bone formation and bone resorption. Defective coupling of bone formation to resorption plays a key role in the pathogenesis of osteoporosis (Reppe, Datta & Gautvik, 2017; Wauquier, Leotoing, Coxam, Guicheux & Wittrant, 2009). Osteoblasts originate from multipotent mesenchymal progenitors, control bone formation not only by regulating bone matrix synthesis and mineralization but also by orchestrating the process of bone resorption (Yeo et al., 2007). Age‐related bone loss is characterized by the reduction of bone formation, which mainly results from the decreased number and activity of osteoblasts (Kassem & Marie, 2011). Apoptosis is thought to be one of the primary regulators of osteoblast homeostasis. Osteoblast apoptosis increases with age (Komori, 2016; Tower, 2015) and is responsible for reduction in osteoblast number and activity, which contribute to decreased bone formation (Jilka & O'Brien, 2016; Jilka, Weinstein, Parfitt & Manolagas, 2007; Weinstein & Manolagas, 2000). Therefore, increased osteoblast apoptosis results in the unbalanced coupling of bone formation and resorption and ultimately leads to bone loss and osteoporosis.

Oxidative stress occurs as a consequence of an imbalance between the formation and inactivation of reactive oxygen species (ROS), which plays roles in aging and associated complications (Rottenberg & Hoek, 2017). Advanced oxidation protein products (AOPPs) are considered to be biomarkers of oxidant‐mediated protein damage. They are a group of carbonyl‐, dityrosine‐, and pentosidine‐containing protein products generated by reaction of plasma proteins with hypochlorous acid and chloramines during oxidative stress and generally considered to be carried mainly by albumin in the blood circulation (Capeillere‐Blandin, Gausson, Descamps‐Latscha & Witko‐Sarsat, 2004; Marsche et al., 2009; Witko‐Sarsat et al., 1996). The accumulation of AOPPs is prevalent in diverse redox‐related diseases, such as rheumatoid arthritis (Wu et al., 2016), inflammatory bowel diseases (Xie et al., 2014), diabetes (Kalousova, Skrha & Zima, 2002), chronic kidney disease (Witko‐Sarsat & Descamps‐Latscha, 1997), and postmenopausal osteoporosis (Wu et al., 2015). In addition to being the products of oxidative stress, AOPPs are also inducers of ROS generation (Witko‐Sarsat et al., 1996, 1998). Previous studies have shown that AOPPs can mediate multiple pathogenic processes by inducing a redox‐dependent apoptosis in diverse cells, such as intestine epithelial cells (Xie et al., 2014), podocytes (Zhou et al., 2012), and chondrocytes (Wu et al., 2016; Ye et al., 2017).

We have previously found that oxidative stress and AOPP levels increased with age, and AOPP accumulation was correlated with age‐related bone loss (Zhang, Zhong, Hou, Jiang & Chen, 2011). Our study has also demonstrated that AOPP treatment aggravated bone loss and deteriorated bone microstructure in the aged rats (Zeng et al., 2014). Here, we demonstrate that AOPPs induce osteoblastic cell apoptosis both in vitro and in vivo. AOPP‐induced osteoblastic cell apoptosis is mainly mediated by the nicotinamide adenine dinucleotide phosphate (NADPH) oxidases‐dependent ROS generation, which activates mitogen‐activated protein kinases (MAPK)‐mediated intrinsic apoptosis pathway.

## 2 RESULTS

### 2.1 AOPPs induced apoptosis in MC3T3‐E1 cells

**Figure 1:**
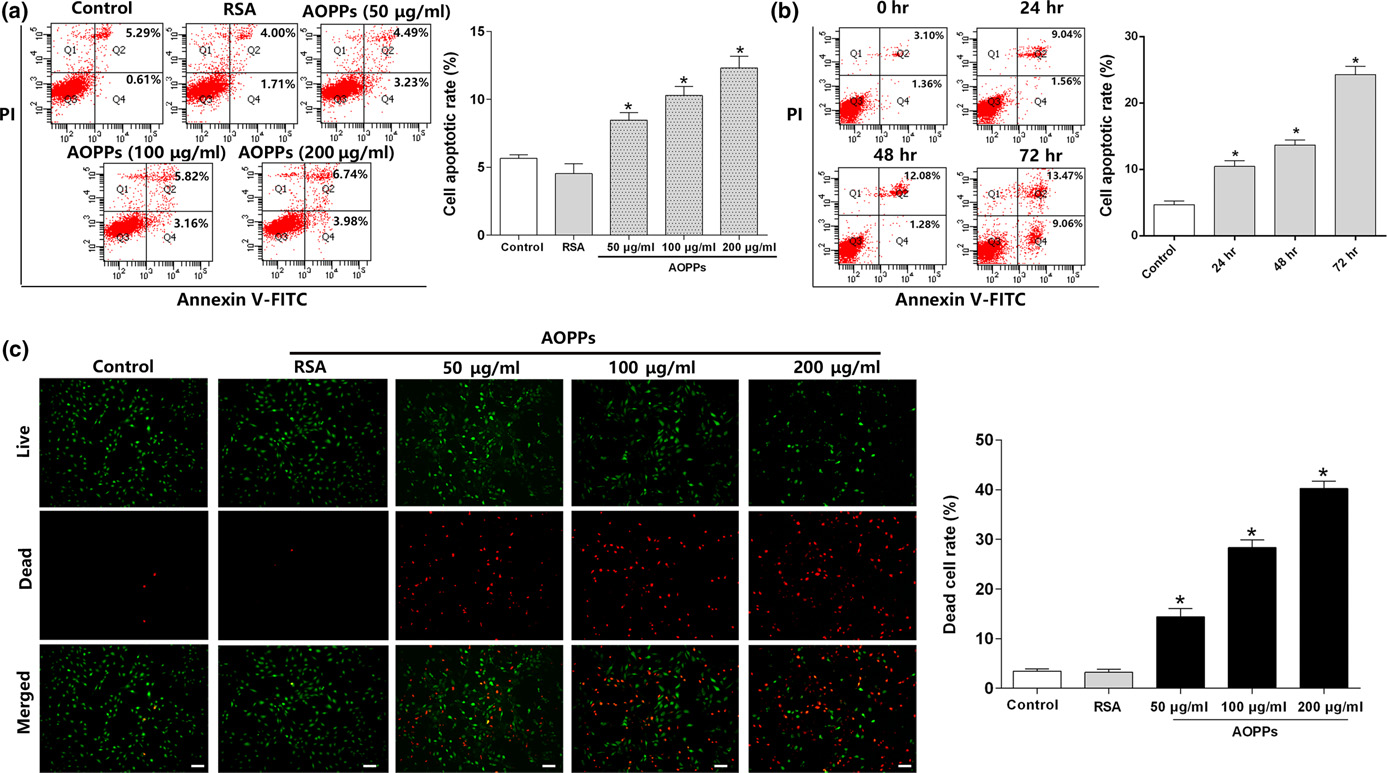
AOPPs induced apoptosis in MC3T3‐E1 cells. (a,b) Flow cytometry assay using Annexin V‐FITC/PI staining showed that AOPP treatment increased apoptosis in MC3T3‐E1 cells in a dose‐ and time‐dependent manner. Three independent technical replicates for each of the flow cytometry experiment were conducted. (c) Fluorescent microscopy analysis using Live and Dead Assay Stain showed AOPP treatment (0–200 μg/ml) for 24 hr increased apoptosis in MC3T3‐E1 cells in a dose‐dependent manner. Live cells stain green, and dead cells stain red (Scale bar = 100 μm). (d) Flow cytometry analysis of Live and Dead Assay Stain showed that the ratio of dead cells increased with the AOPP concentration. Data were presented as mean ± *SD*. *p < .05 vs. control

To clarify whether AOPPs induce apoptosis in MC3T3‐E1 cells, we incubated cells with increasing concentrations (50–200 μg/ml) of AOPPs for 24 hr or 200 μg/ml of AOPPs for increasing times (0–72 hr). Flow cytometry assay indicated that exposure of MC3T3‐E1 cells to AOPPs showed increased apoptosis in a dose‐ and time‐dependent manner (Figure 1a,b), and this was further confirmed by the Live and Dead Assay Stain (Figure 1c,d).

### 2.2 AOPPs induced intracellular ROS generation via the activation of NADPH oxidase

**Figure 2:**
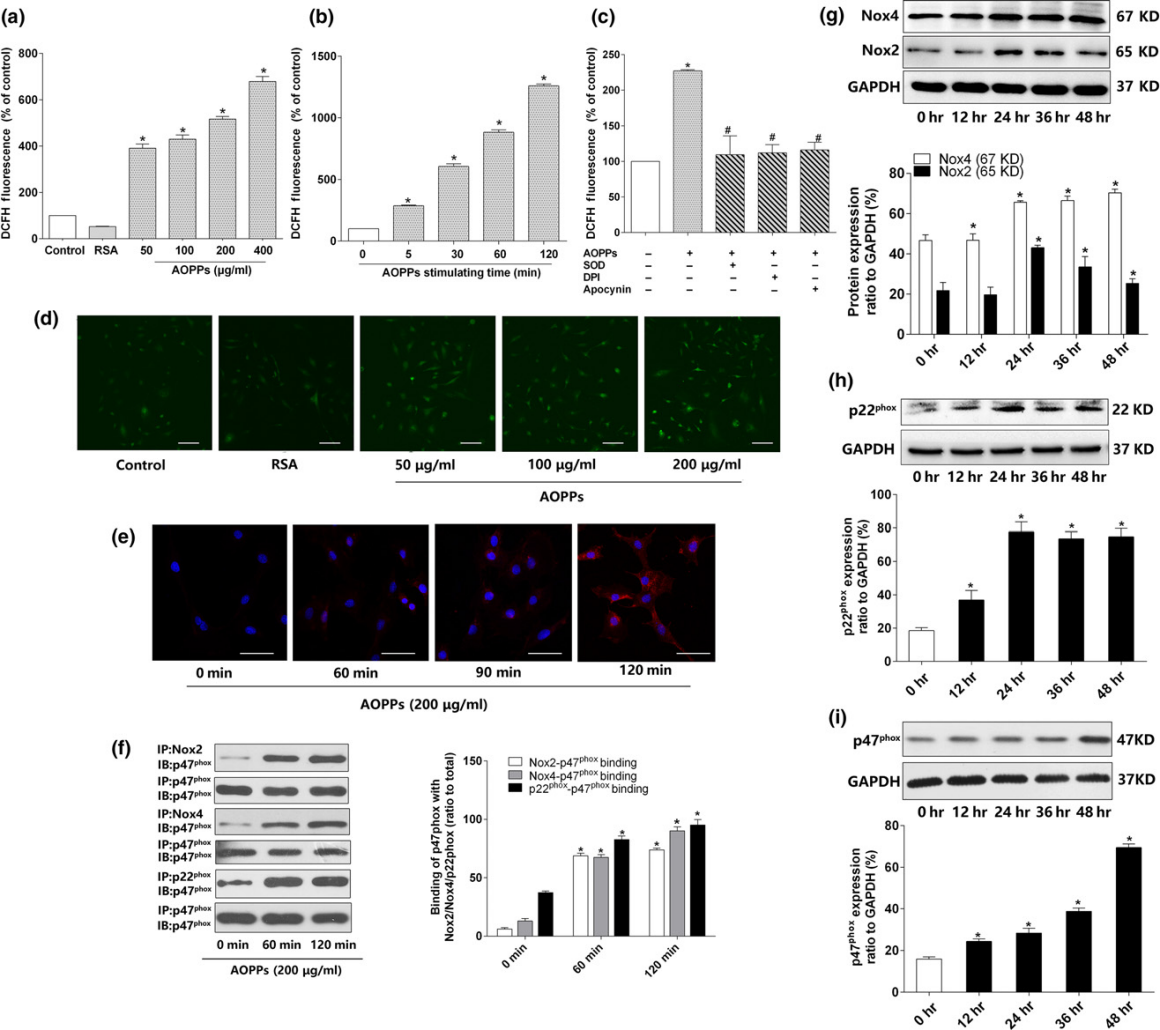
AOPPs increased intracellular ROS generation via the activation of NADPH oxidase. (a) AOPPs (0–400 μg/ml, 120 min) could induce intracellular ROS production in a concentration‐dependent manner. (b) AOPPs (0–120 min, 200 μg/ml) could induce intracellular ROS production in a time‐dependent manner. (c) AOPP‐induced intracellular ROS production was significantly decreased by pretreatment with apocynin (100 μm), DPI (10 μm), and SOD (50 U/ml). (d) Representative confocal microscope images using DCFH‐DA revealed that AOPPs induced ROS production in MC3T3‐E1 cells in a concentration‐dependent manner (Scale bar = 100 μm). (e) Representative confocal microscope images of AOPP‐induced (200 μg/ml) membrane translocation of p47^phox^ in 120 min (Scale bar = 100 μm). (f) Results of coimmunoprecipitation showed that AOPP treatment (200 μg/ml) enhanced binding of p47^phox^ to Nox2, Nox4, and p22^phox^ in 120 min. (g–i) MC3T3‐E1 cells were incubated with AOPPs (200 μg/ml) for different time periods (0–48 hr). Immunoblotting revealed that NADPH oxidase subunits Nox2, Nox4, p22^phox^, and p47^phox^ significantly increased compared with control group. Cells in the inhibitor group were pretreated with apocynin, DPI, and SOD, respectively, for 40 min before AOPP administration, and SOD was present during AOPP incubation. Data were presented as mean ± *SD*. *p < .05 vs. control. #*p* < .05 vs. AOPP group

We previously demonstrated that AOPPs induced excessive ROS production by the NADPH oxidase (Wu et al., 2016; Xie et al., 2014; Zhou et al., 2012). Therefore, we examined the intracellular ROS level in MC3T3‐E1 cells. As shown in Figure 2a,b, AOPP administration significantly increased intracellular ROS generation in a dose‐ and time‐dependent manner, whereas native rat serum albumin (RSA) had no effect. The increased ROS generation was further confirmed by confocal microscopy analysis (Figure 2d). The above‐mentioned effects were also confirmed by the superoxide probe dihydroethidium (DHE) assay (Figure S1).

To verify whether NADPH oxidase is the source of ROS generation, MC3T3‐E1 cells were preincubated with inhibitors of NADPH oxidase. As shown in Figure 2c, the NADPH oxidase inhibitor diphenylene iodonium (DPI) or apocynin significantly inhibited AOPP‐induced ROS generation. Furthermore, the presence of radical scavenger superoxide dismutase (SOD) also significantly decreased ROS level. We further analyzed the activation of NADPH oxidase in AOPP‐treated MC3T3‐E1 cells. As shown in Figure 2e–i, AOPPs induced a rapid membrane translocation of p47^phox^ and promoted the binding of p47^phox^ to the membrane subunits Nox2, Nox4, and p22^phox^ and increased the expression of NADPH oxidase key subunits, such as Nox2, Nox4, p22^phox^, and p47^phox^.

### 2.3 AOPPs activated MAPKs by NADPH oxidase‐dependent ROS generation

**Figure 3:**
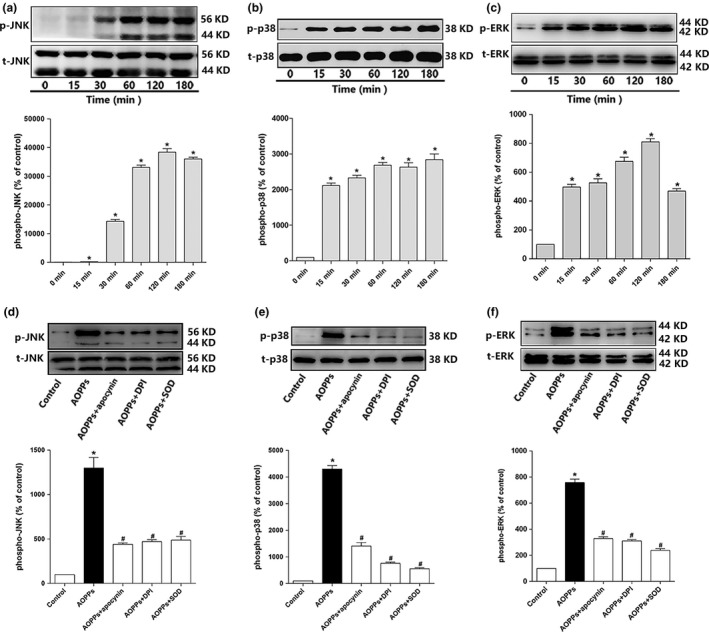
AOPPs activated MAPKs by NADPH oxidase‐dependent ROS generation. (a–c) AOPP (200 μg/ml) treatment significantly increased JNK, p38, and ERK1/2 phosphorylation of MC3T3‐E1 cells in 180 min. (d–f) Apocynin (100 μm), DPI (10 μm), and SOD (50 U/ml) significantly blocked AOPP‐induced phosphorylation of JNK, p38, and ERK1/2. Cells in the inhibitor group were pretreated with apocynin, DPI, and SOD, respectively, for 40 min before AOPP administration, and SOD was present during AOPP incubation. Data were presented as mean ± *SD*. *p < .05 vs. control. #*p* < .05 vs. AOPP group

MAPKs are redox sensitive and have also been shown to be involved in apoptosis in various cell types (Shen & Liu, 2006; Tower, 2015; Cagnol & Chambard, 2010). Therefore, we tested whether AOPPs activated MAPK signaling pathway. As shown in Figure 3a–c, AOPP treatment resulted in a marked increase in phosphorylated JNK, p38, and ERK1/2. While the NADPH oxidase inhibitors DPI or apocynin and the ROS scavenger SOD significantly blocked the activation of MAPKs (Figure 3d–f), which indicated that activation of MAPKs was mediated by NADPH oxidase‐dependent ROS generation.

### 2.4 AOPPs triggered intrinsic apoptosis pathway by NADPH oxidase‐dependent MAPK signaling

**Figure 4:**
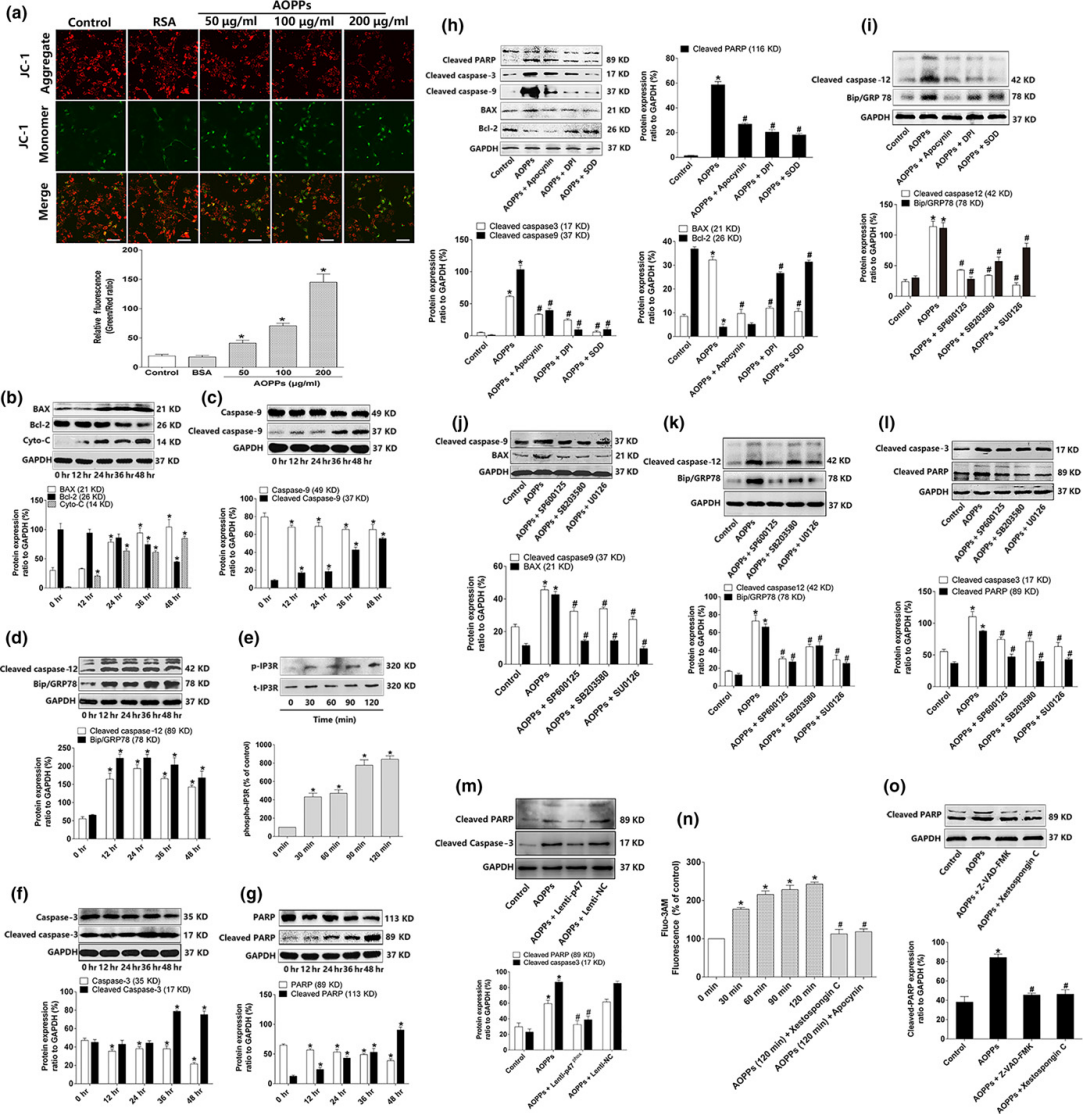
AOPPs triggered intrinsic apoptosis pathway by NADPH oxidase‐dependent, MAPK signaling. (a) Confocal microscopy analysis using JC‐1 staining revealed that AOPP challenge for 24 hr decreased the ▵Ψm level of MC3T3‐E1 cells in a dose‐dependent manner (Bar = 100 μm). Numerical data were expressed in terms of the ratio of JC‐1 aggregates to JC‐1 monomers, the increased ration stand for the decreased ▵Ψm. (b–g) AOPP treatment (200 μg/ml) significantly increased the expression of BAX, cytochrome c, cleaved caspase‐9, cleaved caspase‐12, BiP/GRP78, phosphorylated Ca^2+^ channel IP3R, cleaved caspase‐3, and cleaved PARP, while decreased the expression of Bcl‐2, intact caspase‐9, intact caspase‐3, and intact PARP in 48 hr. (h,i) Pretreated with apocynin (100 μm), DPI (10 μm), and SOD (50 U/ml) significantly decreased AOPP‐induced (200 μg/ml, 48 hr) expression of cleaved caspase‐3, cleaved PARP, cleaved caspase‐9, BAX, cleaved caspase‐12, and BiP/GRP78, while increased the expression National Natural Science Foundation of Bcl‐2. (m) p47^phox^ lentiviral RNAi vector transfection significantly decreased AOPP‐induced expression of cleaved caspase‐3 and cleaved PARP. (j–l) JNK inhibitor SP600125 (10 μm), p38 inhibitor SB203580 (10 μm), and ERK inhibitor U0126 (10 μm) significantly decreased AOPP‐induced (200 μg/ml, 48 hr) expression of cleaved caspase‐9, BAX, cleaved caspase‐12, BiP/GRP78, cleaved caspase‐3, and cleaved PARP. (n) Intracellular calcium level was determined by Fluo‐3/AM. AOPP (200 μg/ml) treatment induced Ca^2+^ overload in a time‐dependent manner, while this effect could be blocked by IP3R inhibitor Xestospongin C (5 μm) and NADPH oxidase inhibitor apocynin (100 μm). (o) Pretreated with caspase inhibitor Z‐VAD‐FMK (20 μm) and IP3R inhibitor Xestospongin C (5 μm) significantly decreased AOPP‐induced (200 μg/ml, 48 hr) cleaved PARP expression. Cells in the inhibitor group were pretreated with apocynin, DPI, SOD, SP600125, SB203580, U0126, Xestospongin C, and Z‐VAD‐FMK, respectively, for 40 min before AOPP administration, and all of them were present during AOPP incubation. Data were presented as mean ± *SD*. *p < .05 vs. control. #*p* < .05 vs. AOPP group

Intrinsic apoptosis pathway is mainly characterized by mitochondria dysfunction and plays a role in the pathogenesis of age‐related disorders (Tower, 2015). Therefore, we examined whether AOPPs triggered intrinsic apoptosis pathway. As shown in Figure 4a, AOPP treatment resulted in the loss of mitochondrial membrane potential (▵Ψm) in a dose‐dependent manner in MC3T3‐E1 cells. Exposure of MC3T3‐E1 cells to AOPPs showed an increased expression of BAX, cytochrome c, cleaved caspase‐9, cleaved caspase‐12, binding immunoglobulin protein (BiP)/glucose‐regulated protein 78 (GRP78), cleaved caspase‐3, cleaved PARP, decreased expression of Bcl‐2, intact caspase‐9, intact caspase‐3 and intact PARP, and increased phosphorylation of inositol 1,4,5‐trisphosphate receptor (IP3R) (Figure 4b–g). AOPP stimulation also increased cytoplasmic Ca^2+^ level (Figure 4n).

We next evaluated the involvement of the NADPH oxidase‐dependent, MAPK signaling axis in AOPP‐induced activation of intrinsic apoptosis pathway. As shown in Figure 4h–l, the pretreatment of NADPH oxidase inhibitors apocynin, DPI, ROS scavenger SOD, JNK inhibitor SP600125, p38 inhibitor SB203580, and ERK1/2 inhibitor U0126 resulted in a decreased expression of cleaved caspase‐9, BAX, cleaved caspase‐12, BiP/GRP78, cleaved caspase‐3, cleaved PARP, and increased expression of Bcl‐2. Furthermore, we also found that p47^phox^ lentiviral RNAi vector transfection significantly decreased the expression of cleaved caspase‐3 and cleaved PARP (Figure 4m). NADPH oxidase inhibitor apocynin and IP3R inhibitor Xestospongin C significantly blocked the elevation of cytoplasmic Ca^2+^ (Figure 4n). Furthermore, caspase inhibitor Z‐VAD‐FMK and IP3R inhibitor Xestospongin C markedly decreased the expression of cleaved PARP (Figure 4o).

### 2.5 AOPPs induced MC3T3‐E1 cell apoptosis through a NADPH oxidase‐dependent, MAPK‐mediated intrinsic apoptosis pathway

**Figure 5:**
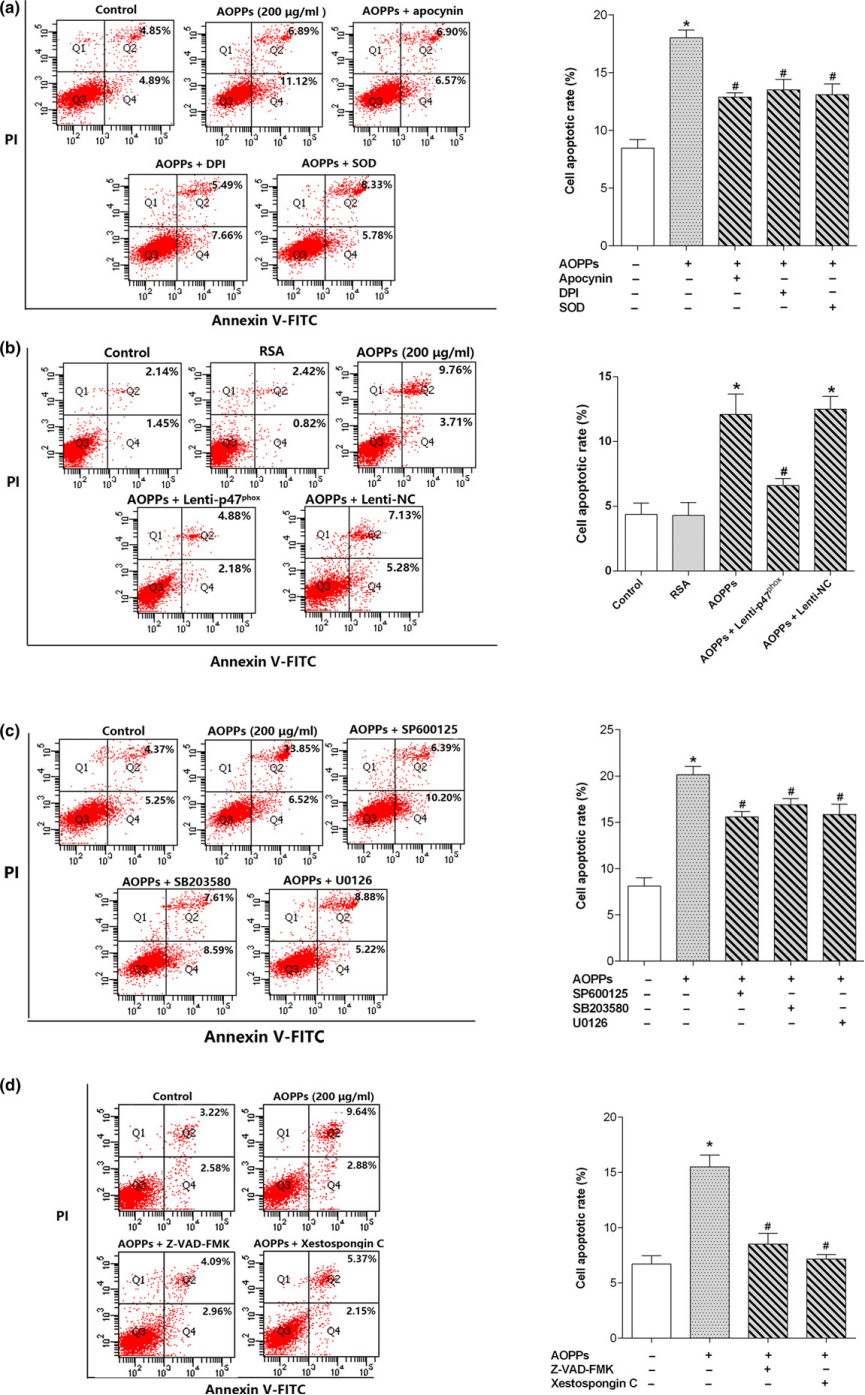
AOPPs induced osteoblast apoptosis through a NADPH oxidase‐dependent, MAPK‐mediated intrinsic apoptosis pathway. (a) Flow cytometry results revealed that AOPP‐induced (200 μg/ml, 24 hr) cell apoptosis could be blocked by preincubating with apocynin (100 μm), DPI (10 μm) and SOD (50 U/ml). (b) Flow cytometry results revealed that p47^phox^ lentiviral RNAi vector transfection markedly decreased AOPP (200 μg/ml, 24 hr)‐induced cell apoptosis. (c) Flow cytometry data indicated that JNK inhibitor SP600125 (10 μm), p38 inhibitor SB203580 (10 μm), and ERK inhibitor U0126 (10 μm) significantly decreased AOPP‐induced (200 μg/ml, 24 hr) cell apoptosis. (d) Flow cytometry results revealed that AOPP‐induced (200 μg/ml, 48 hr) cell apoptosis could be blocked by caspase inhibitor Z‐VAD‐FMK (20 μm) and IP3R inhibitor Xestospongin C (5 μm). Cells in the inhibitor group were pretreated with apocynin, DPI, SOD, SP600125, SB203580, U0126, Xestospongin C, and Z‐VAD‐FMK, respectively, for 40 min before AOPP administration, and all of them were present during AOPP incubation. Three independent technical replicates for each of the flow cytometry experiment were conducted. Data were presented as mean ± *SD*. *p < .05 vs. control. #*p* < .05 vs. AOPP group

To clarify the role of NADPH oxidase‐dependent, MAPK‐mediated intrinsic apoptotic signaling in AOPP‐induced apoptosis, MC3T3‐E1 cells were preincubated with some inhibitors of this signaling axis before AOPP stimulation. As shown in Figure 5a–d, AOPP‐induced apoptosis was significantly blocked by NADPH oxidase inhibitors (apocynin, DPI), ROS scavenger SOD, p47^phox^ lentiviral RNAi vector transfection, MAPK inhibitors (SP600125, SB203580, and U0126), IP3R inhibitor Xestospongin C, and caspase inhibitor Z‐VAD‐FMK. These results indicated that the NADPH oxidase‐dependent, MAPK‐mediated intrinsic apoptosis pathway was involved in AOPP‐induced apoptosis in cultured MC3T3‐E1 cells.

### 2.6 RAGE signaling involved in AOPP‐induced MC3T3‐E1 cell apoptosis

Previous studies have shown that AOPPs could interact with the receptor of advanced glycation end products (RAGE), activate the downstream signaling, and induce cell apoptosis (Wu et al., 2016; Zhou et al., 2012). Therefore, we examined the role of RAGE in AOPP‐induced osteoblast apoptosis. As shown in Figure S2a, FPS‐ZM1, a specific RAGE inhibitor, significantly reduced AOPP‐induced ROS production. Furthermore, FPS‐ZM1 also decreased AOPP‐induced expression of Nox2, Nox4, p22^phox^, and p47^phox^ (Figure S2b–d). We next evaluated the involvement of RAGE in the activation of redox‐sensitive MAPKs. As shown in Figure S2e–g, FPS‐ZM1 markedly blocked AOPP‐induced phosphorylation of JNK, p38, and ERK1/2. Finally, flow cytometry results revealed that FPS‐ZM1 significantly attenuated AOPP‐induced cell apoptosis (Figure S2h).

### 2.7 Chronic AOPP loading induced osteoblast apoptosis and activated NADPH oxidase signaling cascade in aged rats

Intraperitoneal injection of AOPPs daily for 16 weeks markedly increased plasma AOPP level while decreased B‐ALP level (Figure S3a,b).

**Figure 6:**
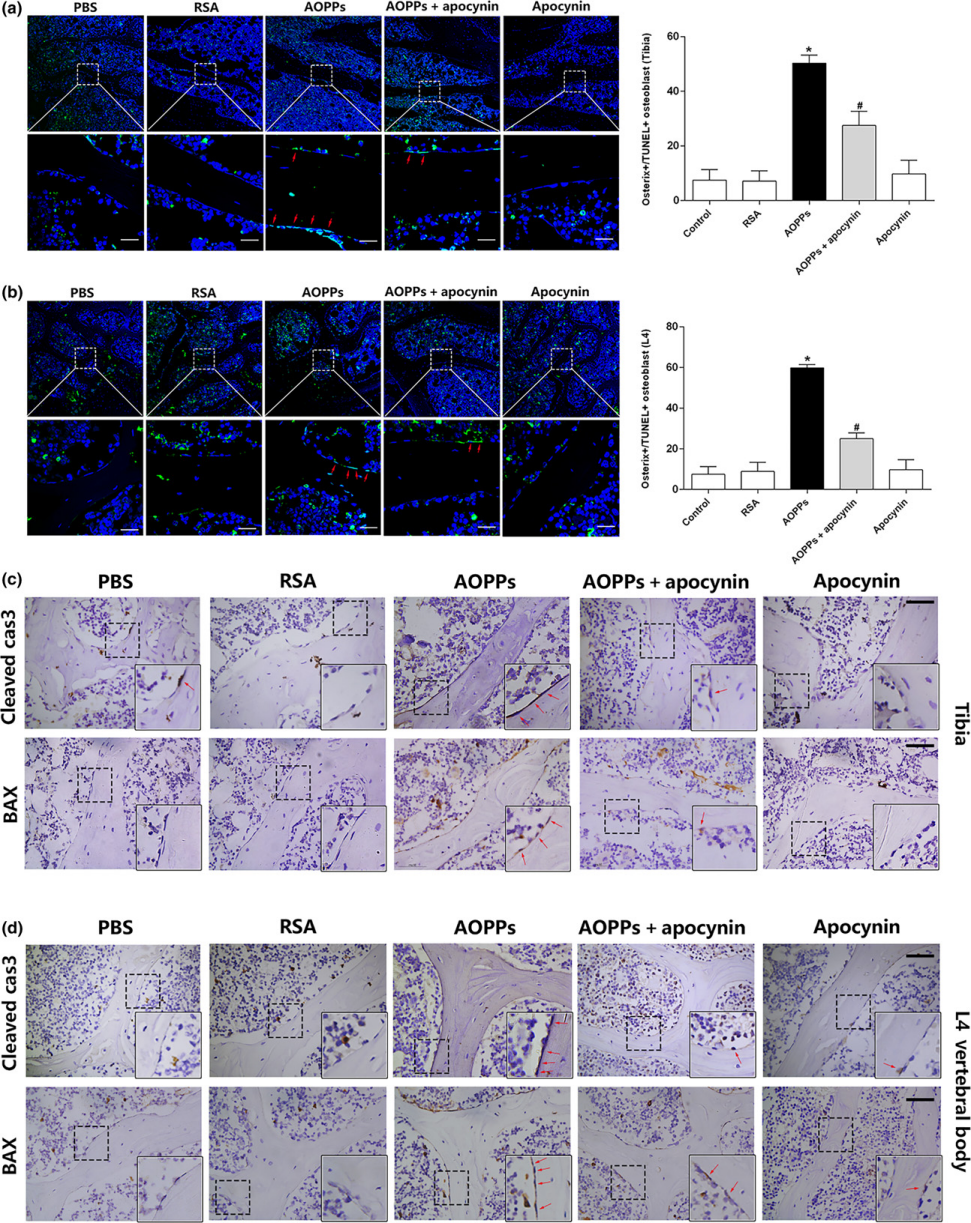
Chronic AOPP loading‐induced osteoblast apoptosis in aged rats. (a,b) Confocal images showed that AOPPs could induce osteoblast apoptosis both in proximal tibias and L4 vertebral bodies in vivo, whereas the effect could be attenuated by apocynin (TUNEL‐positive cells were marked by a red arrow, scale bar = 20 μm). Data were expressed as a % ratio of TUNEL‐positive cells of various groups and mean ± *SD*. *p < .05 vs. control; #*p* < .05 vs. AOPP group. (c,d) Immunohistochemical staining results showed that AOPPs increased apoptosis‐related protein cleaved caspase‐3 and BAX expression in proximal tibias and L4 vertebral bodies at 16 weeks, but were ameliorated by apocynin. Higher magnification of the boxed areas is shown on the bottom to the right, and positive staining is indicated by red arrow (Scale bars represent 50 μm for the main panel)

We conducted TUNEL staining to examine whether AOPP‐induced osteoblast apoptosis occurred in vivo. As shown in Figure 6a,b, chronic AOPP loading‐induced osteoblast apoptosis was demonstrated in both proximal tibias and L4 vertebral bodies. Furthermore, immunohistochemical staining also showed an increased expression of cleaved caspase‐3 and BAX in osteoblasts of AOPP‐challenged rats (Figure 6c,d). We next evaluated whether NADPH oxidase signaling cascade was activated by AOPP in vivo. Immunohistochemical studies showed that chronic AOPP loading resulted in upregulated expression of NADPH oxidase subunits (Nox2, Nox4, p47^phox^, and p22^phox^), increased the phosphorylation of MAPKs (JNK, p38, and ERK1/2) in the osteoblasts of AOPP‐treated rats (Figure S4a–d). Administration of NADPH oxidase inhibitor apocynin significantly attenuated AOPP‐induced osteoblast apoptosis and inhibited the activation of NADPH oxidase signaling cascade (Figure 6a–d and Figure S4a–d).

### 2.8 Chronic AOPP loading deteriorated bone microstructure and accelerated bone loss in aged rats

**Figure 7:**
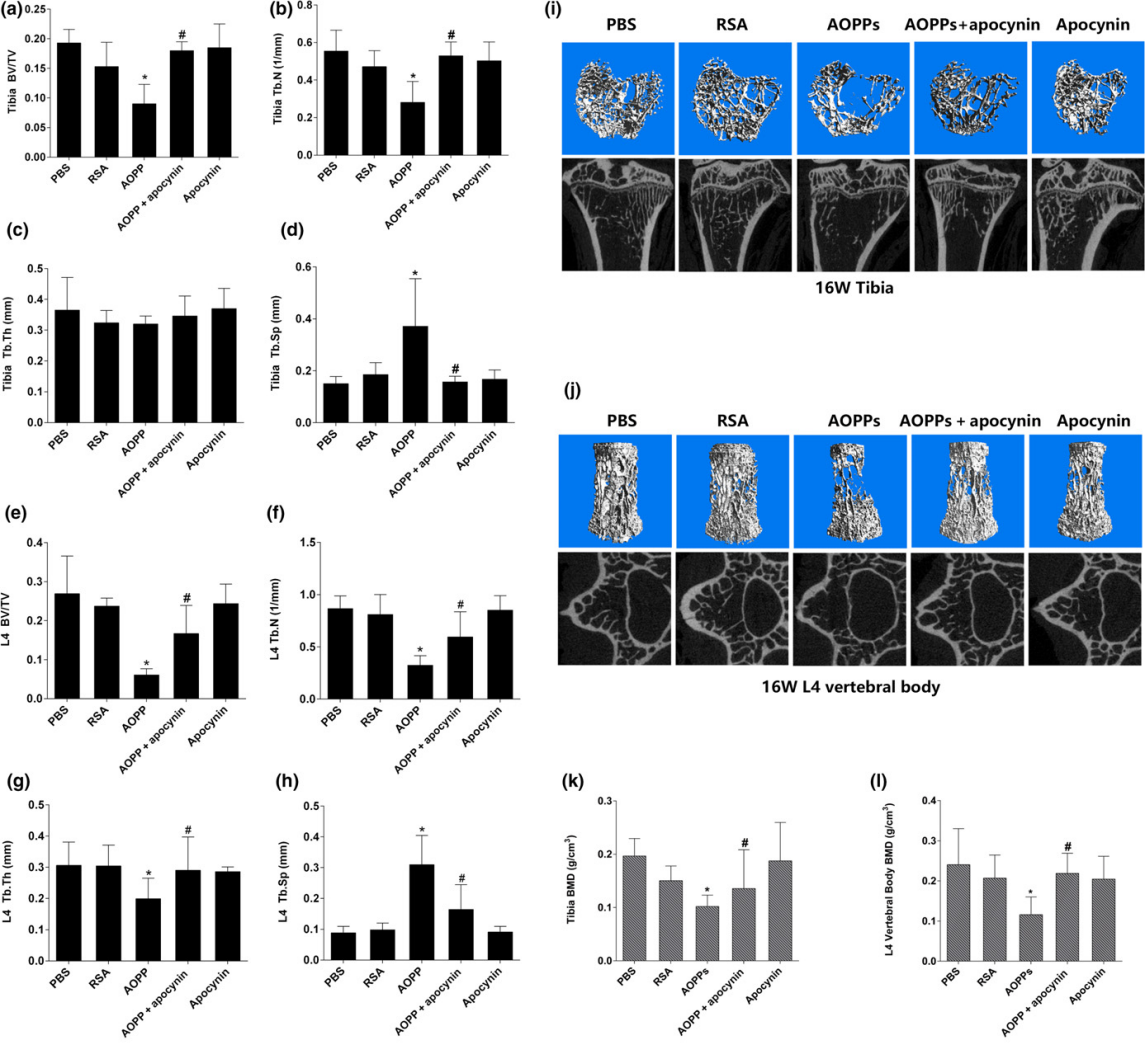
AOPPs deteriorated bone microstructure and accelerated bone loss in aged rats. (a–d) AOPP challenge decreased the BV/TV (Bone Volume/Total Volume), Tb.N (Trabecular Number), but increased Tb.Sp (Trabecular Spacing) in proximal tibias. (e–h) AOPP challenge decreased the BV/TV (Bone Volume/Total Volume), Tb.N (Trabecular Number) and Tb.Th (Trabecular Thickness), but increased Tb.Sp (Trabecular Spacing) in L4 vertebral bodies. (i,j) Micro‐CT three‐dimensional reconstruction and cross‐sectional images showed that AOPP administration caused a severe deterioration to bone microstructure of proximal tibia and L4 vertebral body. (k,l) AOPP accumulation significantly decreased the trabecular BMD (Bone Mineral Density) of the proximal tibias and L4 vertebral bodies. Data were presented as mean ± *SD*. *p < .05 vs. control; #*p* < .05 vs. AOPP group

We then evaluated the effect of AOPPs on bone microstructure and bone mass. Micro‐CT evaluation showed that AOPP challenge decreased the BV/TV and Tb.N, but increased Tb.Sp in proximal tibias (Figure 7a–d). In L4 vertebral bodies, AOPP administration decreased the BV/TV, Tb.N, and Tb.Th while increased Tb.Sp (Figure 7e‐h). This was further confirmed by representative micro‐CT three‐dimensional reconstructions and cross‐sectional images (Figure 7i,j). In addition, we also found that chronic AOPP loading resulted in decreased bone mineral density (BMD) in trabecular of proximal tibias and L4 vertebral bodies in 16 weeks by micro‐CT analysis (Figure 7k,l). Administration of NADPH oxidase inhibitor apocynin alleviated deterioration of bone microstructure and the decrease in BMD caused by AOPP treatment (Figure 7a–l).

## 3 DISCUSSION

AOPPs play a crucial role in the pathological process of diverse diseases (Kalousova et al., 2002; Witko‐Sarsat et al., 1996, 1998; Xie et al., 2014). Under physiological conditions, plasma AOPP concentration is relatively low while increases with aging (Komosinska‐Vassev et al., 2012; Zhang et al., 2011). Osteoporosis is a common skeletal disease in aged and is characterized by low bone mass and microstructure deterioration of bone tissue (Cummings & Melton, 2002; Ford, Bass, Zhao, Bai & Zhao, 2011; Reginster & Burlet, 2006). Numerous studies have proven that osteoporosis was associated with a higher oxidative stress index values and total plasma oxidant status (Almeida & O'Brien, 2013; Sendur, Turan, Tastaban & Serter, 2009; Zhang et al., 2011). Our previous study also demonstrated a positive relationship between AOPP accumulation and bone loss (Zeng et al., 2014). Age‐related bone loss is characterized by reduced bone formation (Kassem & Marie, 2011). Osteoblast apoptosis not only destroys the activity of osteoblast, but also decreases its quantity, which is a key factor of the pathologic mechanism of age‐related bone loss (Jilka & O'Brien, 2016; Jilka et al., 2007; Weinstein & Manolagas, 2000). However, the effect of AOPPs on osteoblast apoptosis remains poorly understood. In the present study, we certified that AOPPs induced osteoblastic cell apoptosis through a NADPH oxidase‐dependent, MAPK‐mediated intrinsic apoptosis pathway.

Osteoblast is a kind of highly specialized cells that produce and mineralize matrix. Excessive osteoblast apoptosis has a close relationship with age‐related bone loss (Jilka et al., 2007; Weinstein & Manolagas, 2000). AOPPs have been identified as a new class of potent inducers of apoptosis. We previously demonstrated that AOPP stimulation caused apoptosis of chondrocytes through a redox‐dependent intrinsic apoptosis pathway in vitro (Wu et al., 2016). In this study, we found that exposure of osteoblastic MC3T3‐E1 cells to AOPPs leads to increased apoptosis. AOPP‐induced apoptosis was further confirmed by the in vivo study, in which TUNEL staining confirmed that chronic AOPP loading caused osteoblast apoptosis in aged rats, which was associated with decreased BMD and deteriorated bone microstructure. Therefore, the increased osteoblast apoptosis might be the pathology basis of AOPP‐induced bone loss or osteoporosis.

It is now widely accepted that ROS at physiological level is beneficial and acts as essential signaling molecule to maintain health and longevity, while excessive ROS production induces oxidative stress and causes damage to cells and organs. NADPH oxidase is one of the main sources of ROS production, and p47^phox^ is an important subunit of NADPH oxidase (Rastogi, Geng, Li & Ding, 2016). A previous study showed knockout of p47^phox^ decreased over 50% of ROS production in p47^phox^ −/− mice compared with the wild‐type mice, which indicated that decrease in physiological ROS level would cause a negative effect to bone metabolism, and finally resulted in bone loss (Chen et al., 2015). It was well demonstrated that AOPPs could induce ROS generation via the activation of NADPH oxidase. (Kalousova et al., 2002; Liu et al., 2006; Weinstein & Manolagas, 2000; Witko‐Sarsat et al., 1996; Wu et al., 2016; Xie et al., 2014). We found that AOPPs could activate the NADPH oxidase and significantly increase ROS production in vitro and in vivo. AOPP stimulation induced more than seven times ROS production in osteoblastic cells, compared with control group. ROS overproduction by AOPP challenge finally resulted in osteoblast dysfunction and bone loss. Apocynin, a NADPH oxidase inhibitor, markedly reversed AOPP‐induced bone loss in aged SD rats. However, we also found that apocynin alone had no effect on the bone mass, which seems to disagree with a previous study, in which apocynin increased bone mass in SAMP6 mouse (Sun et al., 2015). In fact, aged SD rat is a normal senescence model, while SAMP6 mouse is a senescence‐accelerated model. SAMP6 mouse has a widely decrease in the BMD and bone mass, in combination with a significant bone microstructure destruction, compared with the normal mouse at the same age (Takeda et al., 1997). In addition, enhanced effect of apocynin in senescence models was further demonstrated by the in vitro study, in which apocynin promoted the proliferation of bone mesenchymal stem cells (BMSCs) from SAMP6 mouse, but it had no effect on the BMSCs from normal SD rats (Sun et al., 2015).

The intrinsic apoptosis pathway is a form of programed cell death which mainly characterized by mitochondria dysfunction (Shalini, Dorstyn, Dawar & Kumar, 2015; Tower, 2015). In this process, stimulation such as oxidative stress decreases the ▵Ψm, increases expression of BAX, and decreases Bcl‐2, and then results in mitochondrial membrane permeabilization and the releases of cytochrome c into cytoplasm, activates a series of pro‐apoptotic protein, and leads to cell death (Tower, 2015). In our study, AOPP stimulation significantly decreased ▵Ψm of osteoblastic cell and induced a marked expression of pro‐apoptosis protein such as cytochrome c, BAX, and cleaved caspase‐9, which activated the intrinsic pathway. Endoplasmic reticulum (ER) is the primary storage site for intracellular Ca^2+^, which can release Ca^2+^ from the ER Ca^2+^ channel (Scorrano et al., 2003). ER stress acts as a decider in cell life and death, which can be marked by BiP/GRP78. ER stress has a close relationship with intrinsic apoptosis pathway, and in this process, cleaved caspase‐12 induces the cleavage of caspase‐9. ER stress‐related Ca^2+^ overload could be taken up by closely mitochondria, which decrease ▵Ψm. By these steps, ER stress takes part in the intrinsic pathway. In the present study, we proved that AOPP stimulation induced ER stress and related Ca^2+^ over load, which further promoted the intrinsic apoptosis process. Caspase‐3 is an executioner caspase which could be activated by intrinsic apoptosis pathway (Walters et al., 2009). PARP is the main substrate of caspase‐3 and responsible for DNA repairment (Xie et al., 2014). We found that AOPP administration significantly increased the expression of cleaved caspase‐3 and cleaved PARP which finally lead to osteoblast apoptosis. Besides, we also found that caspase inhibitor Z‐VAD‐FMK and IR3R inhibitor Xestospongin C, significantly decreased AOPPs increased apoptosis, which further supported that AOPPs triggered intrinsic apoptosis pathway in osteoblast. Furthermore, we found that the NADPH oxidase inhibitors, ROS scavenger, and MAPK inhibitors could alleviate AOPP‐induced intrinsic apoptosis pathway and cell apoptosis, which suggested that the NADPH oxidase‐dependent, MAPK signaling axis played a dominant role in AOPP‐induced osteoblast apoptosis.

RAGE is a transmembrane receptor of the immunoglobulin superfamily, which is capable of binding many ligands. RAGE was well demonstrated to be the receptor of AOPPs (Wu et al., 2016; Yamamoto & Yamamoto, 2012; Zhou et al., 2012). Interaction of AOPPs and RAGE can activate downstream signal molecules, such as NADPH oxidase and MAPKs, and increase intercellular ROS generation, eventually induce cell apoptosis (Wu et al., 2016; Zhou et al., 2012). In the present study, we found that the high‐affinity RAGE‐specific inhibitor FPS‐ZM1 significantly decreased AOPP‐induced ROS generation, NADPH oxidase subunits upregulation, and MAPK phosphorylation and finally attenuated the apoptosis of osteoblastic cells (Figure S2). Therefore, RAGE signaling cascades are involved in AOPP‐induced osteoblast apoptosis.

In conclusion, our study shows that AOPPs can induce osteoblast apoptosis by the NADPH oxidase‐dependent, MAPK‐mediated intrinsic apoptosis pathway, which may play a very important role in the age‐related bone loss. AOPPs are not only the biomarkers of oxidative stress, but also inducers of ROS generation and oxidative stress. Reducing AOPP generation and its cascading effect may be helpful for treating senile osteoporosis.

## 4 EXPERIMENTAL PROCEDURES

### 4.1 AOPP preparation and determination

AOPPs were prepared according to the procedure described previously (Witko‐Sarsat et al., 1996). In brief, rat serum albumin (RSA, Sigma, St. Louis, MO, USA) solution (20 mg/ml) was incubated with 40 mm hypochlorous acid (Fluke, Buchs, Switzerland) in phosphate‐buffered saline (PBS, pH = 7.4) for 30 min at the room temperature. Prepared samples were dialyzed against PBS to remove free hypochlorous acid. To remove contaminated endotoxin, all samples were passed through a Detoxi‐Gel column (Pierce, Rockford, IL). Endotoxin levels in AOPP–RSA and unmodified RSA were then measured using a Limulus Amoebocyte Lysate kit (Sigma, St Louis, MO) and were found to be below 0.05 ng/mg protein. AOPP content in the sample was determined as described previously (Witko‐Sarsat et al., 1998).

### 4.2 Cell culture

Murine osteoblastic MC3T3‐E1 cells (The Committee of Type Culture Collection, Chinese Academy of Sciences, Beijing, China) were used as in vitro models. The bone‐forming capacity of MC3T3‐E1 cells was identified by the real‐time PCR detection of B‐ALP, RUNX2, and osteocalcin (specific phenotypic markers of osteoblast) and the Alizarin Red S test (Figure S5). MC3T3‐E1 cells were seeded in 25‐cm^2^ flat‐bottom culture flasks and supplemented with αMEM (Gibco, Life Technologies, California, USA) containing 10% fetal bovine serum (FBS, Gibco, Life Technologies, California, USA) at 37°C in a humidified atmosphere with 5% CO_2_. After reaching a subconfluent state, the cells were subcultured, usually 3 days after seeding. All the experiments were performed using passage 6–12.

### 4.3 Real‐time PCR

The TRIzol RNA isolation system (Life Technologies, Grand Island, NY) was used to prepare the total RNA according to the manufacturer's instruction. One microgram of total RNA was reverse transcribed using the PrimeScript™ RT reagent kit with gDNA Eraser (Takara Biotechnology Co.). The primer sequences are as follows: mouse RUNX2, 5′‐CCACAAGGACAGAGTCAGATTACA‐3′ and 5′‐TGGCTCAGATAGGAGGGGTA‐3′; mouse osteocalcin, 5′‐AGACTCCGGCGCTACCTT‐3′ and 5′‐CTCGTCACAAGCAGGGTTAAG‐3′; mouse B‐ALP, 5′‐AAGGCTTCTTCTTGCTGGTG‐3′ and 5′‐GCCTTACCCTCATGATGTCC‐3′.

### 4.4 Determination of cell apoptosis

MC3T3‐E1 cells were cultured in six‐well plates at the concentration of 2 × 10^5^ cells per well. Annexin V‐fluorescein isothiocyanate (FITC)/propidium iodide (PI) double‐staining kit (KeyGen Biotech, China) was used to detect the apoptosis rate. Briefly, cells were collected by trypsinization (without EDTA) and centrifugation, then use the ice‐cold PBS to wash the cells twice and resuspended with 500 μl binding buffer, then 5 μl FITC and 5 μl PI were added into the buffer and incubated in the dark at the room temperature for 15 min. All the cells collected were analyzed by FACSCalibur flow cytometer (Becton Dickinson, USA). Either early apoptosis (Annexin V‐FITC‐positive, PI‐negative) or late apoptosis (Annexin V‐FITC‐positive, PI‐positive) was considered apoptotic cells. Live and Dead Assay Stain (Abcam) was used to distinguish between live and dead cells. Cells were seeded at confocal dishes. After different treatments, all the cells were washed with PBS for three times, incubated with the Live and Dead dye for 10 min at room temperature in the dark. Then cells were observed under the fluorescent microscope (Olympus, Tokyo, Japan). Flow cytometer was also used to analyze the dead cell in the Live and Dead dye tests.

### 4.5 Determination of intracellular ROS generation

Intracellular ROS was detected by the probe 2′,7′‐dichlorofluorescein diacetate (DCFH‐DA). In brief, MC3T3‐E1 cells were incubated with 10 μm DCFH‐DA for 30 min at 37°C. Fluorescence intensity (Ex/Em = 488/525) was measured on a SpectraMax M5 system (Molecular Devices, California, USA). In addition,dihydroethidium (DHE, 5 μm) in combination with flow cytometry was also used to detect ROS production in MC3T3‐E1 cells.

### 4.6 Determination of mitochondrial membrane potential

The fluorescent dye JC‐1 (KeyGen Biotech, China) was used to assess the change in mitochondrial membrane potential. Briefly, cells seeded into the confocal dishes were incubated with the mixture of 1 μl JC‐1 staining fluid and 500 μl incubation buffer in the dark at 37°C for 30 min, then washed with the incubation buffer twice, and observed with the confocal microscope (Olympus, Tokyo, Japan).

### 4.7 Measurement of intracellular Ca^2+^

Intracellular Ca^2+^ was determined by cell‐permeable calcium‐sensitive fluorescent dye Fluo‐3/AM (Beyotime, China). MC3T3‐E1 cells seeded in 96‐well plate were incubated with 5 μm Fluo‐3/AM at 37°C for 30 min. The fluorescence intensity of Fluo‐3/AM probes (Ex/Em = 488/525) was analyzed by SpectraMax M5 system (Molecular Devices, California, USA).

### 4.8 Lentiviral vector infection

The lentiviral vector was purchased from Genomeditech (Shanghai, China). The sequence for specially targeting mouse p47^phox^ was GCCAGCACTATGTGTACATGT (5′–3′); the sequence for not targeting lentiviral vector was TTCTCCGAACGTGTCACGT (5′–3′). For lentiviral transfection, MC3T3‐E1 cells were grown to 30% confluence and then transfected with the lentiviral RANi vector targeting p47^phox^ (Lenti‐p47) or not targeting lentiviral vector (Lenti‐NC) for 72 hr. Western blot result showed that p47^phox^ expression in MC3T3‐E1 cells was significantly decreased after lentiviral vector transfection (Figure S6).

### 4.9 Western Blot

Western blot was used to detect the oxidative stress‐ and apoptosis‐related proteins. Cultured cells were washed with ice‐cold PBS three times and then lysed with RIPA lysis buffer. Supernatant was centrifuged at 4°C, 13,800×g for 30 min to collect the protein. The protein concentration was determined by BCA Protein Assay Kit (Thermo, Life Technologies, California, USA). The samples were separated by SDS‐polyacrylamide gel electrophoresis (PAGE) using 6%–15% acrylamide gels and then transferred to polyvinylidene fluoride (PVDF) membranes (Millipore, Billerica, MA, USA). After incubation with primary and secondary antibodies, the protein bands were detected with chemiluminescence detection reagents (Millipore, Billerica, MA, USA). Antibodies were used as follows: Apoptosis Antibody Sampler Kit (Mouse Preferred),MAPK Family Antibody Sampler Kit, Phospho‐MAPK Family Antibody Sampler Kit, rabbit anti‐BAX, rabbit anti‐Bcl‐2, rabbit anti‐cytochrome c, rabbit anti‐BiP, rabbit anti‐IP3R, rabbit anti‐phospho‐IP3R, and rabbit anti‐GAPDH were all from Cell Signaling Technology (CST, Beverly, MA, USA). Rabbit anti‐cytochrome b245 light chain (p22^phox^), goat anti‐NCF1 (p47^phox^), rabbit anti‐NADPH oxidase 2, and rabbit anti‐NADPH oxidase 4 were from Abcam (Abcam,Cambridge,UK). Goat anti‐rabbit and rabbit anti‐goat IgG‐horseradish peroxidase (HRP) secondary antibodies were from Abcam (Abcam,Cambridge,UK). Integrated density of all the protein bands was analyzed with Image J software.

### 4.10 Immunofluorescence detecting p47^phox^ membrane translocation

MC3T3‐E1 cells were seeded in confocal dishes with different treatments, fixed with paraformaldehyde, and permeabilized with 0.2% Triton‐X. After blocking with 5% BSA, cells were incubated with rabbit anti‐p47^phox^ overnight at 4°C. After washing and incubating with Cy3‐conjugated anti‐rabbit IgG (Beyotime, China), the cells were stained by DAPI (Abcam,Cambridge,UK). The location of p47^phox^ in MC3T3‐E1 cells was observed by confocal microscope (Olympus, Tokyo, Japan).

### 4.11 Immunoprecipitation

Immunoprecipitation was used to detect the combination of p47^phox^ to p22^phox^, Nox2 and Nox4. In this process, the cell lysates were preincubated with Dynabeads Protein G (Novex, Invitrogen) and incubated with anti‐p47^phox^ antibody, then tested by Western blot with anti‐Nox2 antibody, anti‐Nox4 antibody, and anti‐p22^phox^ antibody to determine the combination rate.

### 4.12 Animal studies

All the animal studies were approved by the Laboratory of Animal Care and Use Committee of Southern Medical University. Forty male SD rats aged 18 months with the initial weight 594–672 g were housed in a standard environment with regular light/dark cycles and free access to water and chow diet. All of the rats were randomly divided into five groups containing eight rats per group and received the following treatments: group 1, daily intraperitoneal injection of vehicle (PBS, pH = 7.4); group 2, daily intraperitoneal injection of unmodified RSA (50 mg/kg); group 3, daily intraperitoneal injection of AOPPs (50 mg/kg); group 4, daily intraperitoneal injection of AOPPs (50 mg/kg) together with apocynin (NADPH oxidase inhibitor) at 100 mg/kg in drinking water and changed the water daily to quantify the volume of water intaking; and group 5, apocynin at 100 mg/kg/day in drinking water. At the end of 16 weeks, animals were anesthetized with isoflurane and sacrificed. Then collected blood samples from abdominal aorta puncture, after centrifuging (980×g, 4°C) for 20 min, the supernatant was separated and stored in −80°C for further study. The fourth lumbar vertebrae (L4) and left tibias were collected and fixed in 4% paraformaldehyde. Eight rats were analyzed per group.

### 4.13 Blood AOPPs and Bone‐specific alkaline phosphatase (B‐ALP) concentration

The method of determining AOPP levels in plasma was the same as the method used for AOPP content determination in the AOPP–RSA compound. Bone‐specific alkaline phosphatase (B‐ALP) level of serum was determined by ELISA kit (CUSABIO, China).

### 4.14 Micro‐CT analysis

Micro‐computed tomographic (Micro‐CT) examination was carried out using the Sky Scan 1176 CT scanner system (Aartselaar, Belgium). All the samples were scanned at a nominal resolution (pixels) of 18 μm with source voltage of 80 kV and source current of 313 μA. Reconstruction was carried out using the SkyScan NRecon software. The volume of interest (VOI) was defined as approximately 1.2 mm from growth plate and extended from this position for a further 2.0 mm of the proximal tibia. As for the vertebral body, the trabecular bone region was outlined for each micro‐CT slice, excluding both the cranial and caudal endplate regions. Series of two‐dimensional image data were gathered to reconstruct the three‐dimensional images using the CTvox software. The bone mineral density (BMD) of the trabecular of tibias and L4 vertebral bodies and parameters of the trabecular bone microstructure, such as bone volume over total volume (BV/TV), trabecular thickness (Tb.Th), trabecular number (Tb.N), and trabecular spacing (Tb.Sp), were calculated using the CTAn software.

### 4.15 TUNEL assay

To explore whether the AOPPs could cause osteoblast apoptosis in vivo, status of osteoblast apoptosis was detected by TUNEL staining (indicative of DNA fragmentation) in decalcified bone sections of various treatment groups in 16 weeks. Briefly, all the tibias and L4 vertebral bodies of each group were fetched out and fixed in 4% paraformaldehyde, then decalcified with 10% EDTA for 3 weeks and embedded with paraffin, and sliced into 5‐μm‐thick transverse sections following the standard method. The apoptotic cells in bone tissue were assessed with deoxynucleotidyl transferase‐mediated nick end labeling assay (TUNEL assay, Roche, Mannheim, Germany). Sections were also treated with DAPI for 15 min at room temperature which were used as a nuclear counterstain. Finally, the sections were rinsed several times in PBS, mounted on cover slips in FluorSave mounting medium, and visualized under ZEISS LSM 880 (Carl Zeiss, Germany).

### 4.16 Immunohistochemical staining

Paraffin‐embedded bone tissue was sliced into 5‐μm‐thick transverse sections. After the process mentioned above, slices were incubated with primary antibodies against Nox2, Nox4, p22^phox^, p47^phox^, cleaved caspase‐3, BAX, phospho‐JNK, phospho‐p38, and phospho‐ERK1/2 at 4°C overnight. The immunostaining was examined with a Leica DM5000 B (Leica, Germany).

### 4.17 Statistical analysis

All the experiments were repeated at least three times. Continuous variables were presented as mean ± *SD*. One‐way ANOVA analysis was used to detect differences among groups. Two‐tailed *p*‐value of < .05 was considered statistically significant. Statistical analyses were conducted with SPSS 20.0 software (SPSS Inc, Chicago, IL).

## AUTHORS' CONTRIBUTION

Zhao‐Ming Zhong, Si‐Yuan Zhu, and Jian‐Ting Chen designed the study. Si‐Yuan Zhu performed the majority of the study. Qian Wu provided technical support. Jing‐Shen Zhuang, Zhong‐Yuan Liu, Cong‐Rui Liao, and Shi‐Gan Luo helped performed the in vivo study. Si‐Yuan Zhu analyzed the data. Si‐Yuan Zhu wrote the manuscript.

## CONFLICT OF INTEREST

Authors declare no conflict of interest.
