# Supplementary material for: Advanced oxidation protein products induce pre‐osteoblast apoptosis through a nicotinamide adenine dinucleotide phosphate oxidase‐dependent, mitogen‐activated protein kinases‐mediated intrinsic apoptosis pathway
Source: Aging Cell. 2018 Apr 16;17(4):e12764. doi: 10.1111/acel.12764 (PMC6052394; doi:10.1111/acel.12764)
Supplement: Supplementary file 1 [file ACEL-17-na-s001.pdf]

Advanced oxidation protein products induce pre-osteoblast apoptosis through a NADPH oxidase-dependent, MAPKs-mediated intrinsic apoptosis pathway

Si-Yuan Zhu, Jing-Shen Zhuang, Qian-Wu, Zhong-Yuan Liu, Cong-Rui Liao, Shi-Gan Luo, Jian-Ting Chen and Zhao-Ming Zhong

SUPPORTING INFORMATION

Fig. S1 AOPPs induced ROS production detected by Dihydroethidium (DHE).

Fig. S2 RAGE signaling involved in AOPPs-induced MC3T3-E1 cell apoptosis.

Fig. S3 AOPPs administration increased the plasma AOPPs level and decreased the plasma B-ALP level.

Fig. S4 Chronic AOPPs loading activated NADPH oxidase signaling cascade in aged rats.

Fig. S5 Bone-forming capacity of murine osteoblastic MC3T3-E1 cells.

Fig. S6 Silencing $p47^{phox}$ with lentiviral RNAi vector transfection.

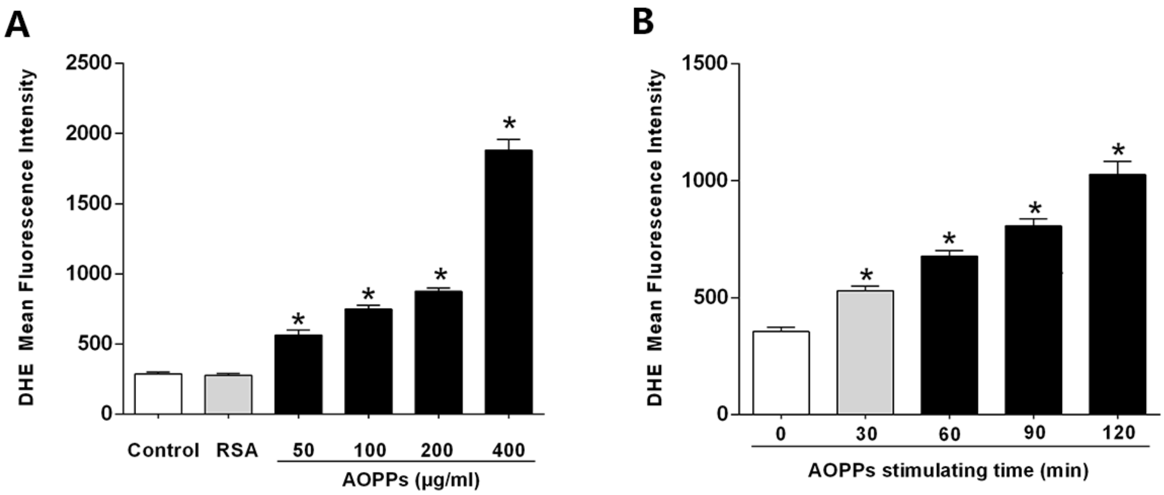

Fig. S1 AOPPs induced ROS production detected by Dihydroethidium (DHE). (A) AOPPs (0-400μg/ml, 120min) could induce intracellular ROS production in a concentration- dependent manner. (B) AOPPs (0-120min, 200μg/ml) could induce intracellular ROS production in a time- dependent manner. Data were presented as mean ± SD. * P<0.05 versus control.

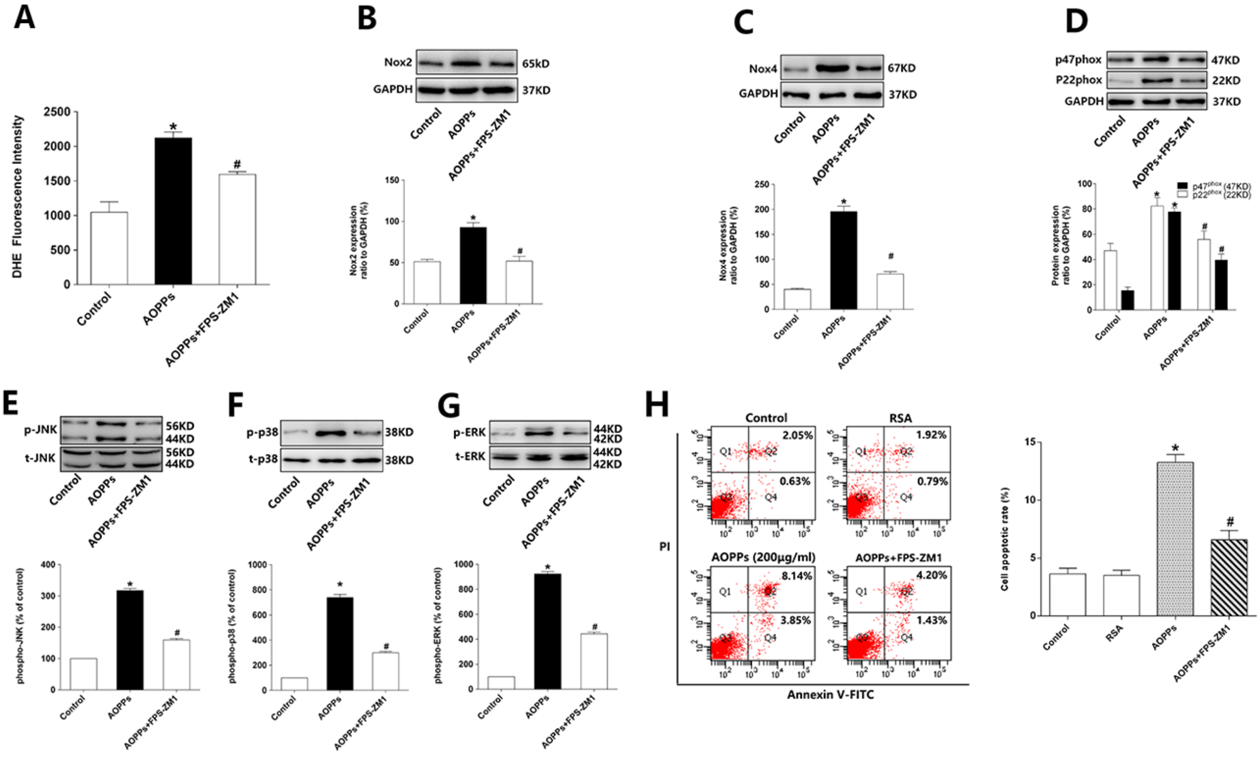

Fig. S2 RAGE signaling involved in AOPPs-induced MC3T3-E1 cell apoptosis. (A) AOPPs-induced (200μg/ml, 120 min) intracellular ROS production was significantly decreased by RAGE specific inhibitor FPS-ZM1. (B-D) AOPPs-induced (200μg/ml, 48h) increased expression of NADPH oxidase subunits (Nox2, Nox4, $p47^{phox}$, $p22^{phox}$) was significantly suppressed by RAGE specific inhibitor FPS-ZM1. (E-G) RAGE specific inhibitor FPS-ZM1 significantly blocked AOPPs-induced (200μg/ml, 180min) phosphorylation of JNK, p38 and ERK1/2. (H) Flow cytometry revealed that AOPPs-induced (200μg/ml, 24h) cell apoptosis could be attenuated by pre-incubation with FPS-ZM1. Cells in the inhibitor group were pre-treated with FPS-ZM1 (5μM) for 40min before AOPPs administration. FPS-ZM1 was present during AOPPs incubation. Data were presented as mean ± SD. * $P<0.05$ versus control; # $P<0.05$ versus AOPPs group.

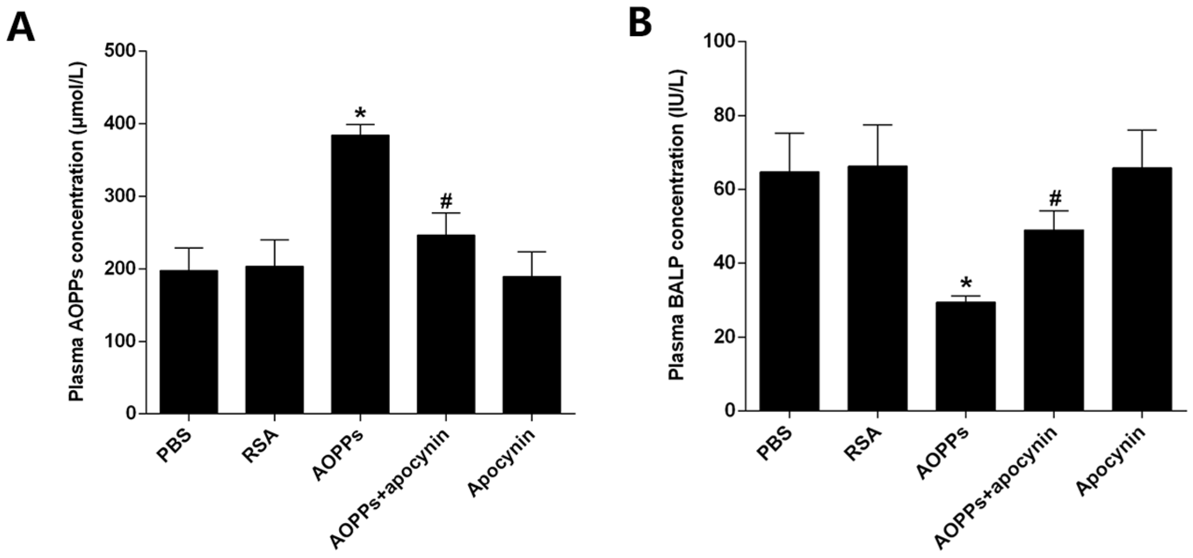

Fig. S3 AOPPs administration increased the plasma AOPPs level and decreased the plasma B-ALP level. (A) Intraperitoneal injection of AOPPs significantly increased plasma AOPPs concentration, while apocynin could attenuate the effect. (B) Intraperitoneal injection of AOPPs presented a significant low level of plasma B-ALP concentration, apocynin intervention markedly reduced the influence of AOPPs. Data were presented as mean ± SD. * $P<0.05$ versus control; # $P<0.05$ versus AOPPs group.

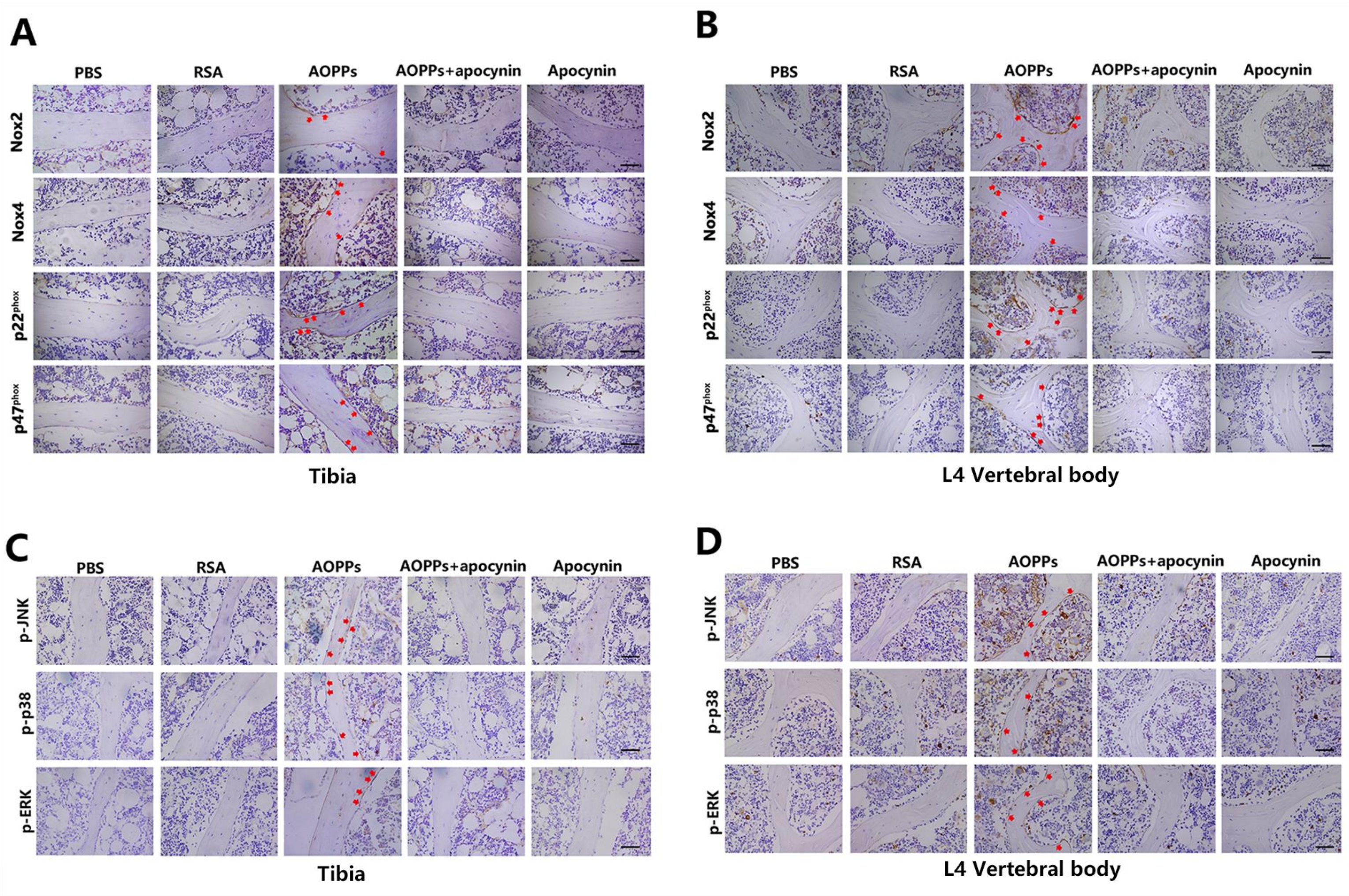

Fig. S4 Chronic AOPPs loading activated NADPH oxidase signaling cascade in aged rats. (A-B) Immunohistochemical staining of proximal tibias and L4 vertebral bodies showed that AOPPs administration (50mg/kg) significantly increased Nox2, Nox4, $p47^{phox}$, $p22^{phox}$ expression. While apocynin could attenuate this effect (Scale bar=50μm). (C-D) Immunohistochemical staining of proximal tibias and L4 vertebral bodies showed that AOPPs administration (50mg/kg) significantly induced MAPKs family activation (p-JNK, p-p38, p-ERK1/2). While apocynin could attenuate this effect (Scale bar=50μm).

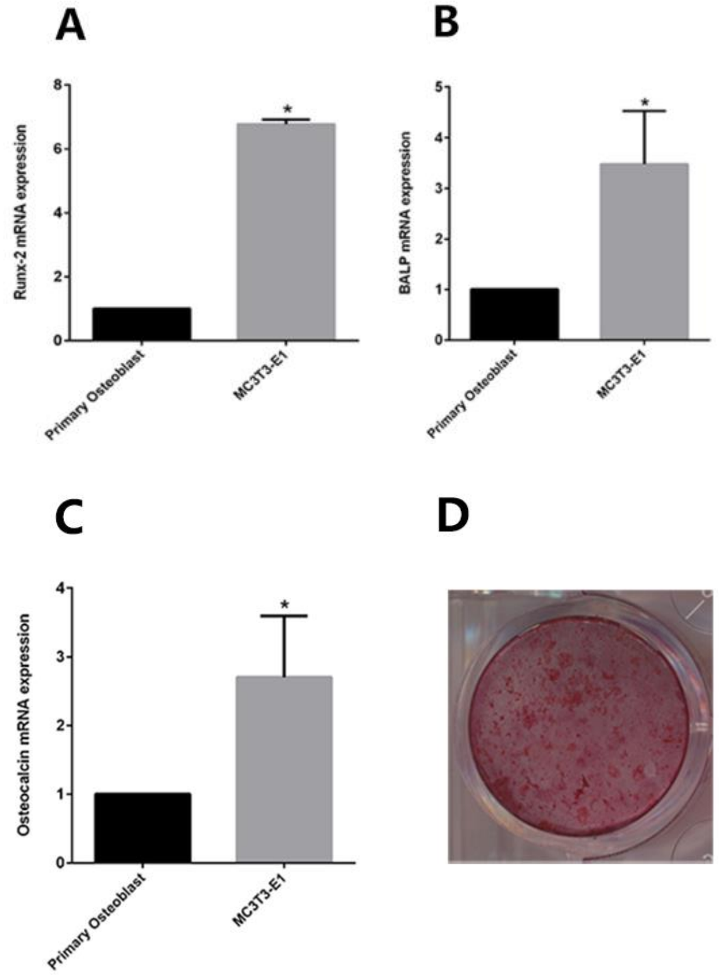

Fig. S5 Bone-forming capacity of murine osteoblastic MC3T3-E1 cells. (A-C) Expression of B-ALP, RUNX2 and osteocalcin genes were shown as highly elevated in comparison with primary osteoblasts obtained from the calvarias of neonatal rats. Data were presented as mean ± SD. * $P<0.05$ versus primary osteoblasts. (D) MC3T3-E1 cells were stained positive by Alizarin Red S after 3 weeks of osteogenic induction.

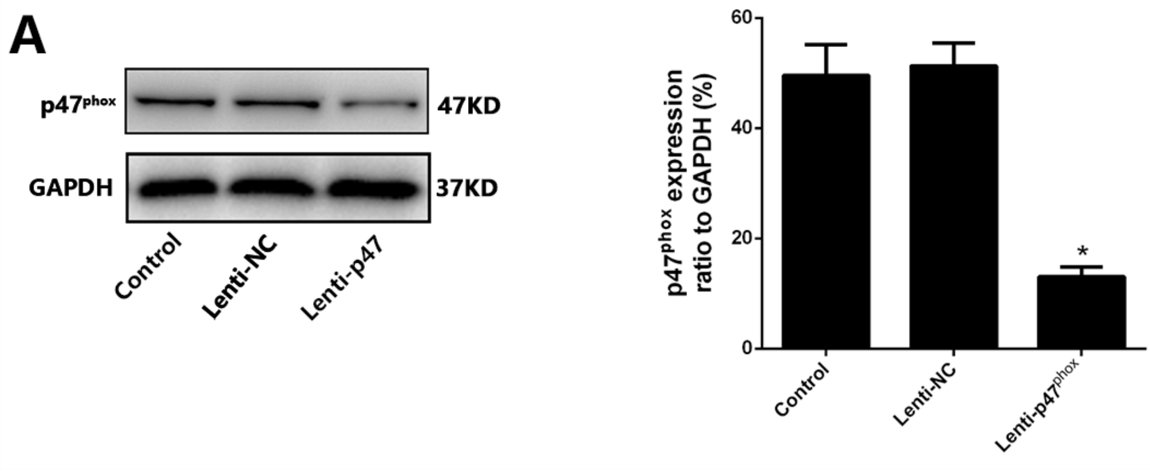

Fig. S6 Silencing p47^{phox} with lentiviral RNAi vector transfection. (A) MC3T3-E1 cells were transfected with a lentiviral RNAi vector targeting p47^{phox} or a lentiviral-negative control vector for 72h. Western blot analysis showed that lentiviral RNAi vector transfection significantly decreased p47^{phox} expression in MC3T3-E1 cells. Data were presented as mean ± SD. * $P<0.05$ versus control.
